# Artificial Intelligence in Ophthalmology: Current Status, Challenges, and Future Perspectives

**DOI:** 10.1002/hcs2.70052

**Published:** 2026-03-06

**Authors:** She Chongyang, Tao Yong

**Affiliations:** ^1^ Department of Ophthalmology Beijing Chao‐Yang Hospital Capital Medical University Beijing China

**Keywords:** artificial intelligence, disease diagnosis, ophthalmology, prognosis prediction, rehabilitation, surgery

## Abstract

Artificial intelligence (AI) is revolutionizing ophthalmology by providing innovative solutions for disease screening, diagnosis, personalized treatment, and the delivery of global healthcare services. This review explores the current state of AI research and applications in the field of ophthalmology, and the applications of telemedicine as well as clinical trials demonstrating AI's utility in reducing diagnostic time and improving treatment outcomes. It also analyzes the challenges faced in practical applications, including economic constraints, data privacy issues, and regulatory hurdles. The review highlights the future directions of AI integration, including highly accurate AI diagnosis, the formulation of personalized treatment plans, AI‐assisted surgical treatment, wearable medical devices, and telemedicine connectivity technologies. In addition, collaborative efforts among academia, the industry, and policymakers are crucial for overcoming these obstacles and fully unleashing the potential of AI in achieving equitable, efficient, and personalized ophthalmic healthcare services.

## Graphical abstract

Current research of artificial intelligence (AI) in ophthalmology.

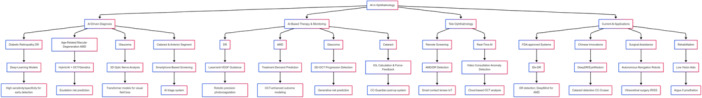

## 1 Background

Ophthalmology, as a visually reliant medical specialty, is uniquely positioned to benefit from advancements in artificial intelligence (AI) and digital technology. Recent developments in deep learning (DL) and convolutional neural networks (CNNs) have transformed image analysis, enabling automated diagnosis and risk prognostication for diseases such as diabetic retinopathy (DR) and glaucoma [1]. Concurrently, telemedicine has reduced healthcare disparities, particularly in underserved regions [2]. Despite progress in automated screening and AI‐driven disease forecasting [3], challenges such as high costs, regulatory complexities, and data privacy concerns hinder widespread implementation [4]. This review examines the current state of AI in ophthalmology, emphasizing innovations, challenges, and future directions, with a focus on presenting reviewing results and evidence synthesis, and conducting critical analysis and comparison of existing studies.

## 2 Literature Search and Screening

This review comprehensively examines the current state, challenges, and future perspectives of AI in ophthalmology. To ensure a comprehensive and up‐to‐date analysis, a systematic search strategy was employed across multiple academic databases, including MEDLINE and EMBASE. The search was configured to retrieve relevant studies published within the time frame from January 1, 2010, to March 1, 2025.

The search was designed to retrieve a wide range of relevant studies. Keywords used in the search included “artificial intelligence,” “deep learning,” “convolutional neural networks,” “ophthalmology,” “diabetic retinopathy,” “age‐related macular degeneration,” “glaucoma,” “cataract,” “ophthalmic therapy,” “tele‐ophthalmology,” and related terms. These keywords were carefully selected to cover various aspects of AI applications in ophthalmology, including disease diagnosis and treatment, telemedicine, and remote monitoring.

The inclusion criteria for studies were broad, encompassing research articles, clinical trials, review papers, and case reports that focused on AI applications in ophthalmology. Studies were included if they investigated the use of AI in disease screening, diagnosis, treatment, prognosis prediction, or the development of related technologies such as tele‐ophthalmology and wearable devices in the field of ophthalmology. Only studies published in English were considered to ensure the quality and comprehensibility of the review.

Exclusion criteria were also defined to refine the search results. Articles that did not directly address AI applications in ophthalmology, such as those focusing solely on general AI concepts without ophthalmic relevance, were excluded. Additionally, conference abstracts without full‐text publications were not included in the review to ensure that only well‐established and peer‐reviewed research was considered.

The selected studies were then critically evaluated based on several factors. Methodological rigor was a key consideration, including aspects such as study design, sample size, and data collection methods. The quality of the AI algorithms described in the studies, including their accuracy, sensitivity, and specificity, was also carefully assessed. Furthermore, the clinical relevance of the research, such as its potential impact on patient care and the practical application of the findings in real‐world clinical settings, was taken into account. By following this systematic approach, this review aims to provide a reliable and comprehensive overview of the role of AI in ophthalmology.

A total of 3105 articles were included in this review after the systematic search and selection process. The majority of the studies focused on AI applications in disease diagnosis (*n* = 1480), followed by treatment and monitoring (*n* = 61), tele‐ophthalmology and remote monitoring (*n* = 170), surgical assistance (*n* = 131), rehabilitation (*n* = 20), and other related areas (*n* = 1243). The evidence synthesis shows that AI has demonstrated promising performance in various aspects of ophthalmology, with high accuracy, sensitivity, and specificity in many diagnostic and prognostic tasks. However, there are significant variations in the quality and generalizability of the studies, with some suffering from small sample sizes, lack of external validation, and potential bias in data collection.

## 3 Current Research of AI in Ophthalmology

### 3.1 Diagnostic Innovations

#### 3.1.1 DR

AI models for DR diagnosis continue to advance. A study utilizing DL architectures and image preprocessing techniques achieved a sensitivity of 93.86% and specificity of 96% in detecting referable DR using a diverse dataset of retinal images [5]. This DL system achieved high accuracy with purely non‐mydriatic inputs, simplifying screening in resource‐limited settings. Its performance is comparable to prior automated DR detection systems developed using mydriatic images, such as Gulshan et al.'s system, which reported 90.3% sensitivity and 98.1% specificity on the EyePACS‐1 dataset [6]. However, this DL system was specifically designed for challenging community‐acquired images by non‐technical field workers. Bellemo et al.'s study in Zambia reported 99.42% sensitivity for vision‐threatening DR, focusing on severe DR detection [7].

Moreover, the development of AI models for DR lesion segmentation has been growing rapidly. Most contemporary segmentation models rely on CNNs, which offer better generalization, automated feature extraction, and greater robustness against image quality variations compared to traditional machine learning [8, 9]. Generative adversarial networks have also been employed in retinal lesion segmentation tasks [8]. Integrating lesion detection into DR screening systems.

#### 3.1.2 Age‐Related Macular Degeneration (AMD)

In the realm of AMD, researchers aim to enhance diagnostic precision through multimodal AI integration. Schmidt‐Erfurth et al. developed an AI model using optical coherence tomography (OCT) and genetic data to predict the progression of AMD, attaining an AUC of 0.68–0.80 in predicting the onset of neovascular and non‐neovascular AMD [10]. Furthermore, Yan et al. used a DL algorithm to predict the progression of late AMD based on color fundus photographs and genotypes, achieving an AUC of 0.85–0.86 [11]. This study combines fundus images with 52 AMD‐related genetic variants, employs a modified CNN to predict the progression of late AMD, enhances accuracy by fusing multimodal data, supports individualized risk assessment, and aids in precise prevention. Banerjee et al. proposed a hybrid sequential prediction model incorporating OCT imaging features and demographic data to predict the risk of exudation in AMD patients, with high AUC values of 0.96 and 0.97 for short‐term and long‐term predictions, respectively [12]. It is suitable for resource‐limited settings and optimizes follow‐up frequency.

#### 3.1.3 Glaucoma

In glaucoma research, efforts have been focused on developing more advanced AI algorithms for early detection and accurate prediction of disease progression. Asaoka et al. employed a DL algorithm trained on macular OCT images to detect early‐onset glaucoma, demonstrating remarkable performance in early diagnosis [13]. Notably, their focus on macular regions differentiates from conventional optic nerve head‐centric approaches, offering a novel anatomical perspective. Li et al. utilized a large longitudinal dataset incorporating spectral‐domain OCT images and comprehensive patient clinical histories to analyze the three‐dimensional structure of the optic nerve head and peripapillary retinal layers, achieving an AUC of 0.92 in predicting glaucomatous progression [14]. Their integration of structural imaging with clinical data contrasts with Asaoka's single‐modality focus, enhancing predictive depth through multi‐source information. Thompson et al. proposed a DL variational autoencoder model trained with a vast amount of visual field data from a large cohort of patients to predict the rates of change and future visual field observations. The model effectively identified a significant proportion of eyes with progressing glaucoma, outperforming traditional methods [15], highlighting a functional data‐driven approach that complements the structural imaging‐based strategies of the other two studies.

#### 3.1.4 Cataract and Anterior Segment Diseases

Recent studies have shown significant advancements in the diagnosis of cataracts and other anterior segment eye diseases using AI. Cheung et al. introduced a computer‐aided diagnosis system using slit‐lamp images [16]. And Wu et al. demonstrated robust performance in cataract diagnostic AI using slit‐lamp photographs [17]. Li et al. achieved a high accuracy of 98.4%–99.8% in diagnosing and grading cataracts using a DL algorithm on slit‐lamp images [18]. Ueno et al. developed an AI algorithm using smartphone images to diagnose cataracts and multiple corneal diseases; the model got high AUC values: 0.998 for normal eyes, 0.986 for infectious keratitis, 0.992 for cataracts [19]. Triage of referral suggestions via smartphone images performed well, with 1.00 sensitivity and specificity for “urgent” cases [19]. This system offers a more accessible alternative for resource‐constrained settings.

Nevertheless, research in the AI diagnosis area has limitations. The study by Damian and Nicoară highlighted the need for more consistent results due to confounding factors from databases, sample sizes, and methods [20]. Additionally, the lack of standardized reporting guidelines and the “black box” nature of some AI algorithms pose challenges to clinical implementation. Future research is expected to address these limitations and improve the accuracy and applicability of AI systems.

### 3.2 AI in Ophthalmic Therapy and Monitoring

#### 3.2.1 DR Treatment

AI plays a crucial role in monitoring the progression or regression of DR by comparing successive retinal images. By utilizing imaging data and patient information, AI can predict the likelihood of disease progression and develop personalized treatment plans [21]. AI models also analyze patient data, including OCT imaging, to predict responses to anti‐vascular endothelial growth factor (VEGF) therapy in diabetic macular edema [22]. AI can also help determine the most effective treatment regimen, including the frequency and timing of anti‐VEGF injections, laser therapy, or combination therapy [23].

Furthermore, AI‐driven robotic technology can assist surgeons in performing delicate surgeries with higher precision. A study by Romanelli et al. demonstrated that laser technology advancements, such as pattern scanning laser photocoagulation, offer more accurate and less harmful treatment options [24].

#### 3.2.2 Personalized AMD Treatment

In AMD treatment, AI integration is a promising avenue for personalized medicine. Gallardo et al. developed a machine learning model achieving mean AUCs of 0.79 for predicting low and high treatment demand in neovascular AMD eyes [25]. Fu et al. used a DL algorithm to predict visual outcomes in neovascular AMD patients [26]. Their study of 926 treatment‐naïve eyes found the algorithm could predict future visual acuity. The *R* ^2^ values were 0.79 (MAE 5.0) after three injections and 0.63 (MAE 7.2) 12 months post‐baseline. Han et al. used a deep neural network to predict anatomical improvement post‐anti‐VEGF treatment [27]. Their research on 2068 images from 517 Korean patients showed the AI model outperformed ophthalmologists in predicting outcomes, with a sensitivity of 0.915, specificity of 0.426, and accuracy of 0.820. Adding OCT images after the first and second injections enhanced the model's predictive capabilities.

Thus, AI holds substantial potential to address diverse clinical needs in AMD management, from optimizing treatment strategies to enhancing prognostic accuracy and supporting personalized patient care.

#### 3.2.3 Glaucoma Treatment and Monitoring

In glaucoma treatment, AI has emerged as a powerful tool for monitoring patients. Mariottoni et al. devised a DL‐augmented method for detecting glaucoma progression within spectral‐domain OCT scans, achieving an AUC of 0.94 [28]. Hussain et al. proposed a DL framework steered by a generative algorithm for predicting glaucoma progression, attaining an AUC of 0.83 [29]. Hou et al. employed a gated transformer network to prognosticate visual field deterioration using longitudinal OCT data [30]. Tian et al. applied discriminative and generative vision transformers for detecting glaucoma progression and predicting Humphrey visual field changes [31]. However, further research is warranted to translate these promising AI‐driven insights into actionable, personalized treatment strategies that can be seamlessly integrated into clinical practice for glaucoma management.

#### 3.2.4 Cataract Management

AI is a valuable tool in cataract management. Pre‐operatively, it's used for cataract detection, grading, and intraocular lens (IOL) power calculation. Ladas et al. showed supervised learning algorithms combined with traditional formulas can improve IOL power calculation accuracy [32]. Notably, pre‐operative AI applications lean heavily on integration with established clinical formulas to enhance precision, whereas intra‐operative systems prioritize real‐time decision support through dynamic data streams like video feeds and sensor inputs. Intra‐operatively, AI helps with surgical phase recognition and workflow analysis. Systems like DeepPhase and Touch Surgery Enterprise analyze cataract surgery videos for phase identification, aiding training and OR efficiency [33]. Auditory force‐feedback sensors alert surgeons during membrane peeling [34]. Post‐operatively, AI predicts complications like posterior capsule opacification and IOL dislocation. Jiang et al. TempSeq‐Net can accurately predict posterior capsule opacification progression [35]. Mohammadi et al.'s prototype ANN predicts capsulotomy‐needed opacification with 87% accuracy and 0.71 AUC [36]. Ghamsarian et al. use CNNs to assess IOL dislocation risk [37].

In pediatric cataract postoperative care, Long et al. developed the “CC‐guardian” AI platform [38]. This platform uses Bayesian and DL algorithms to forecast patients at elevated risk of postoperative complications and to arrange follow‐up visits based on risk stratification. Notably, CC‐Guardian demonstrates superior performance in predicting visual axis opacification and elevated intraocular pressure compared to adult‐focused models. While adult AI models primarily focus on single‐complication prediction, CC‐Guardian's telehealth module further enables intervention decisions with exceptional accuracy by incorporating both follow‐up images and intraocular pressure data. Additionally, CC‐Guardian's dispatching module dynamically adjusts visit timings based on risk stratification, reducing unnecessary visits by 25.3% in real‐world tests.

Although AI shows potential in disease management, its application still faces challenges. The limited availability of public datasets restricts algorithm development and validation [39], and the lack of standardized DL metrics makes it difficult to compare different algorithms. To fully realize AI's potential in improving disease management, further research and validation are needed. Future studies should focus on expanding datasets, incorporating multi‐modal data, conducting long‐term follow‐up, and improving algorithm interpretability.

### 3.3 Tele‐Ophthalmology and Remote Monitoring

#### 3.3.1 Remote Screening

Tele‐ophthalmology aims to overcome geographical barriers in eye care. Mansberger et al. compared telemedicine with a non‐mydriatic camera to in‐person monitoring for DR screening [40]. The telemedicine group had a higher screening rate in the first year (94% vs. 56%, *p* < 0.001), but 20.5% needed further evaluation due to poor image quality. Shekhawat et al. found that routine fundus photography with tele‐consultation in South Indian clinics increased referrals for posterior segment diseases [41]. Kawaguchi et al.'s meta‐analysis indicated that tele‐ophthalmology had a 14% higher disease‐detection odds for AMD and DR [42]. Overall, tele‐ophthalmology can be effective, but image quality and management need improvement.

Mansberger's study highlights telemedicine's superiority in screening reach, while Shekhawat's work underscores its value in resource‐poor regions—though both reveal image quality as a universal bottleneck, more critical in low‐resource settings with limited imaging equipment.

#### 3.3.2 Internet of Things (IoT) and Wearable Devices

In IoT device integration, current research focuses on enhancing the capabilities and interoperability of wearable sensors and other remote monitoring devices. Wearable sensors, such as smart contact lenses, are being developed with more advanced biosensing capabilities to continuously monitor physiological parameters like intraocular pressure, glucose levels, and biomarkers in tear fluid [43]. IoT‐enabled ophthalmic imaging devices, like OCT devices, can automatically upload images to the cloud for AI analysis. However, challenges such as data privacy and security, interoperability, and regulatory frameworks need to be addressed for seamless integration.

#### 3.3.3 Real‐Time Consultation

For real‐time telemedicine consultations, researchers are developing AI algorithms to perform real‐time video analysis during consultations to assist ophthalmologists in detecting subtle ocular abnormalities [44]. These algorithms can detect changes in pupil size, eye movement patterns, and the appearance of the anterior segment [45]. Additionally, AI‐powered chatbots and virtual assistants are being investigated to improve patient communication and triage [46].

## 4 Current Applications

### 4.1 Disease Diagnosis and Screening

AI has become a powerful tool in ophthalmology for disease diagnosis, screening, and enabling personalized medicine. The US Food and Drug Administration (FDA)‐approved IDx‐DR system has demonstrated exceptional diagnostic capabilities, with sensitivity and specificity rates exceeding 96.8%, comparable to experienced ophthalmologists [47, 48]. It can accurately identify the presence or absence of referable DR, facilitating large‐scale screening and timely management, especially in regions with a high prevalence of diabetes and limited access to specialized care.

Google's DeepMind project has collaborated with the UK National Health Service to develop a diagnostic model for the early identification of DR and AMD by AI [49]. Not only are large Internet companies involved, but many local hospitals have also begun introducing AI technology and collaborating with AI providers to deploy AI systems for the screening and diagnosis of fundus diseases. Especially when dealing with a large number of screening images, the AI system demonstrates high efficiency and accuracy.

Thanks to the strong support of national policies and the active investment of technology enterprises, the digital transformation in the field of ophthalmology in China is accelerating. Domestic researchers are actively developing efficient AI algorithms for the screening and diagnosis of ophthalmic diseases. They are mainly applied in DR diagnosis (such as the DeepDR system, EyeWisdom system, ZZ‐EYE‐CDS system, etc.), glaucoma diagnosis (such as the MY‐YD‐01 system and glaucoma prediction model, etc.), and cataract diagnosis (such as the CC‐Cruiser system and AI‐Slitlamp system, etc.) [38, 50].

### 4.2 Tele‐Ophthalmology and Remote Monitoring

Telemedicine and AI algorithms enable patients to enjoy more customized and continuous medical services, and at the same time, the diagnosis and treatment process of doctors becomes more efficient with the assistance of technology [51, 52]. In China, the rapid development of telemedicine enables patients to communicate with ophthalmologists via video at home and obtain professional medical advice. Specifically, there are 5G remote diagnosis, 5G remote fundus laser treatment, 5G remote ophthalmic consultation, and 5G remote micron‐level ophthalmic surgical robots [53]. The popularization of these telemedicine services has improved the efficiency of the use of medical resources.

### 4.3 Surgical Assistance

The application of AI in ophthalmic surgery is just beginning. In vitreoretinal surgery, Nespolo et al. developed an AI‐guided surgical ecosystem [54]. In cataract surgery, Nespolo et al. and Morita et al. focused on identifying critical surgical phases in cataract surgery videos, providing a foundation for AI‐based surgical tools [55, 56]. Wang et al. proposed a DL‐based platform for intelligent cataract surgery supervision and evaluation, offering warnings when surgical phases are missed or skipped [57].

Rahimy et al. and Wilson et al. worked on the development of robot‐assisted intraocular surgery, including the mechanical design and evaluation of the IRISS system [58, 59]. Kim et al. explored autonomous navigation of surgical tools and the combination of deep imitation learning with optimal control for autonomous eye surgery, laying the groundwork for fully autonomous surgical procedures [60, 61].

### 4.4 Rehabilitation

AI technology also plays a significant role in the field of ophthalmic rehabilitation. The OrCam MyEye device captures text and objects through a camera mounted on glasses and then conveys the information through headphones, helping users read text and recognize daily items [62]. In the treatment of amblyopia in children, the AI‐assisted amblyopia treatment system for children uses AI to track eye movements and provide personalized vision training to improve vision. The US FDA‐approved Argus II and the European CE‐certified Retina Implant Alpha AMS enable patients to use AI software to understand new visual signals [63, 64]. ﻿Table 1 summarizes the role of AI in ophthalmology, as mentioned above.

**Table 1:** Summary of AI applications in ophthalmology.

Domain	Current research	Current applications	Clinical impact and advancements	Limitations and challenges
DR	Deep learning for DR diagnosis (sensitivity 93.86%, specificity 96% for referable DR). DL systems on ultra‐widefield photos for vision‐threatening DR and macular edema (AUC > 0.90). CNNs and GANs for DR lesion segmentation.	FDA‐approved IDx‐DR system (sensitivity > 96.8%). Chinese AI systems for DR diagnosis. Tele‐ophthalmology for DR screening.	Early detection and treatment can reduce the risk of vision loss. Large‐scale screening in diabetes‐prevalent areas. Non‐specialist‐friendly screening.	Database, sample, and method‐related inconsistencies. Lack of standardized reporting. “Black box” algorithms. Dataset bias affecting specific populations.
AMD	Multimodal AI for diagnostic precision. AI models predicting AMD progression (AUC: 0.68–0.97).	Clinical AI diagnosis (94%/91% accuracy with OCT/OCTA, 96% with multimodal). Chinese AI systems for AMD diagnosis.	Personalized treatment planning. Early detection for vision preservation.	Multimodal data complexity. Inaccurate prediction models. Lack of standardized metrics. Dataset bias.
Glaucoma	Advanced AI algorithms for early detection and progression prediction (using OCT, visual field data). Deep learning variational autoencoder models for visual field prediction.	Clinic‐based AI glaucoma detection. Chinese MY‐YD‐01 and other models. AI‐assisted progression monitoring.	Early intervention to delay progression. Automated and objective disease management.	Overfitting in algorithms. Ancestry‐related accuracy differences. Weak early‐stage progression models. Poor disease mechanism understanding.
Cataract and anterior segment diseases	AI for cataract diagnosis (98.4%–99.8% accuracy). AI‐based IOL power calculation. Automated screening for corneal and anterior segment issues.	Pre‐operative cataract detection and IOL calculation. Intra‐operative phase analysis. Post‐operative complication prediction. Chinese CC‐Cruiser and AI‐Slitlamp systems.	Improved surgical outcomes. Enhanced surgical efficiency and safety. Early complication prevention.	Limited public datasets. Lack of deep‐learning metrics. Low generalizability of models.
Ophthalmic therapy and monitoring	AI for DR treatment (monitoring, predicting response, treatment planning). Machine learning for AMD treatment demand and visual outcomes. Deep learning for glaucoma progression detection. AI‐based cataract management.	Robotic assistance in DR surgery. Personalized AMD treatment. Glaucoma treatment adjustment. “CC‐guardian” for pediatric cataract follow‐up.	Effective treatment plans. Reduced surgical risks. Better patient prognosis.	Limited data for algorithm development. Lack of evaluation metrics. Difficulties in clinical integration.
Tele‐ophthalmology and remote monitoring	Comparing telemedicine and in‐person DR screening. IoT‐enabled devices and sensors for monitoring. AI for real‐time video analysis in consultations.	Chinese 5G‐enabled tele‐ophthalmology services. AI‐powered chatbots for patient communication.	Remote access to eye care. Comprehensive patient data collection. Efficient tele‐ophthalmology consultations.	Image quality issues. Data privacy and security concerns. IoT interoperability problems. Lack of regulatory frameworks.

Abbreviations: 5G, 5th generation mobile communication technology; AI, artificial intelligence; AMD, age‐related macular degeneration; AUC, area under the curve; CNN, convolutional neural network; DL, deep learning; DR, diabetic retinopathy; FDA, Food and Drug Administration; GANs, generative adversarial networks; IOL, intraocular lens; IoT, internet of things; OCT, optical coherence tomography; OCTA, optical coherence tomography angiography.

### 4.5 Clinical Trails

Global collaboration is critical for AI development in ophthalmology, with shared datasets and standardized trial protocols being essential to address inequities in AI access [65]. The integration of AI in ophthalmology requires rigorous clinical validation to ensure safety, efficacy, and generalizability. While AI models have demonstrated promising performance in retrospective studies, prospective clinical trials remain critical to translate these advancements into real‐world practice.

Few randomized controlled trials (RCTs) have evaluated AI systems in ophthalmology, with most studies focusing on diagnostic accuracy rather than patient outcomes. A randomized study (ClinicalTrials.gov NCT03240848) involving 350 participants demonstrated that the CC‐Cruiser system achieved cataract diagnosis and treatment determination accuracies of 87.4% and 70.8%, respectively. Those were significantly lower than the rates observed with senior consultants, while reducing diagnostic time to 2.79 versus 8.53 min for consultants [66].

Another RCT evaluated an automated DL system for classifying DR in retinal fundus images. It showed high accuracy (AUC ≥ 98%, sensitivity ≥ 95%, specificity ≥ 90%) in international and Mexican datasets, outperforming average ophthalmologist performance, and significantly improved clinicians' sensitivity when used as an assistive tool [67].

The FDA‐approved IDx‐DR system, the first autonomous AI diagnostic tool for DR, underwent a pivotal RCT involving 900 patients across 10 primary care sites (ClinicalTrials.gov NCT02963441) [68]. While it achieved high sensitivity (87.2%) and specificity (90.7%), long‐term studies are needed to confirm its impact on vision preservation and healthcare resource allocation. Additionally, several RCTs are undergoing or about to be launched.

### 4.6 Challenges and Limitations

The deployment of AI in ophthalmology faces several challenges. First, obtaining an adequate quantity of training data is difficult. Hospitals struggle with data organization and sharing due to regulatory, privacy, ethical, and legal concerns, while computer science companies lack access to clinical data despite their computational expertise [69]. Second, the appropriate definition of ground truth and labeling is a significant hurdle. Most algorithms rely on subjective grading by experts of images from medical devices, leading to misclassification and insufficient label information due to varying expert opinions [70, 71]. Third, accurately assessing the accuracy of AI algorithms is crucial. Overfitting is a common problem, often resulting from flaws in the training set design. Prospective clinical trials are gradually becoming the standard for evaluating AI algorithms [6, 72, 73]. Finally, security issues in AI deployment cannot be ignored. Strengthening the security and robustness of AI systems is crucial to preventing potential misdiagnoses and risks in clinical applications.

### 4.7 Future Perspectives

In the future, ophthalmic AI research should prioritize addressing unmet clinical needs, such as improving diagnostic sensitivity and specificity for early‐stage diseases and developing multimodal AI networks for comprehensive disease management [74, 75]. For instance, integrating color fundus photography, OCT, and genetic data could enhance the accuracy of AMD progression prediction [11]. Advanced segmentation networks may also improve the localization of anatomical structures (e.g., optic discs) in fundus images, enabling more robust detection of glaucomatous optic neuropathy [76, 77].

Prospective clinical trials are essential to validate whether AI tools can improve patient outcomes beyond diagnostic accuracy. For example, the IDx‐DR system has shown promise in DR screening, but long‐term studies are needed to confirm its impact on vision preservation [68, 75]. International collaboration and standardized reporting guidelines (e.g., CONSORT‐AI, STARD‐AI) will be critical to address data bias and ensure transparency in AI model development [78, 79].

Moreover, overcoming the “AI black box” challenge through interpretable models (e.g., hierarchical DL systems for glaucoma diagnosis) will be vital to gain clinician trust and facilitate regulatory approval [77, 80]. As highlighted by recent studies, the combination of technical innovation, clinical validation, and ethical frameworks will drive AI toward widespread clinical adoption in ophthalmology [75]. Ethical considerations, including reducing health inequalities and ensuring unbiased algorithms trained on multi‐ethnic datasets, must be integrated into AI design [81, 82]. ﻿Table 2 summarizes challenges and future perspectives in ophthalmic AI, as mentioned above.

**Table 2:** Summary of challenges and future perspectives in ophthalmic AI.

Challenges	Solutions	Future perspectives
Dataset bias (ethnicity, age, comorbidities), limited external validation, and underrepresentation in training data.	Synthetic data augmentation, ethnicity‐agnostic metrics, and post‐market surveillance.	Global collaboration to build diverse datasets and regulatory mandates for transparency in training data composition.
Lack of transparency and explainability in “black box” algorithms.	Integration of interpretable AI models, attention mechanisms for better lesion description, and regulatory requirements for explainability.	Federated learning for diverse training data and interactive interfaces for clinician‐AI collaboration.
Unclear responsibility and liability in case of AI‐related errors, liability fragmentation, and low positive predictive value in some AI‐assisted systems.	Shared liability models (e.g., IDx‐DR's contractual approach), workflow integration with authentication, and regulatory error attribution protocols.	Collaborative governance models with developer explainable failure modes, clinician documentation, and institutional audits.
Privacy concerns in data use and sharing, re‐identification risks, and regulatory fragmentation.	Federated learning, blockchain‐based audits, dynamic consent platforms, and synthetic data generation.	Adaptive frameworks balancing innovation and privacy, with standardized international regulations.
Inconsistent informed consent requirements, algorithmic transparency issues for patients, and difficulties in offering non‐AI alternatives.	Standardized consent frameworks, multilingual AI tools for consent materials, and simplified yet comprehensive explanations.	Integration of AI‐specific disclosures into existing workflows.
Unequal access to AI‐enabled eye care, performance disparities in marginalized groups, and infrastructure‐related barriers.	Device‐agnostic models, culturally adapted training, regulatory incentives, and public–private partnerships.	Proactive equity frameworks, federated learning for global data pooling, and improved digital infrastructure.
Regulatory hurdles for autonomous diagnostic tools, lack of standardized validation across devices and populations.	Clearer regulatory guidelines, prospective clinical trials, and standardized reporting guidelines (e.g., CONSORT‐AI, STARD‐AI).	Regulatory frameworks mandating transparent reporting of training data and failure mode analyses.
Overfitting in AI algorithms due to flaws in the training set design.	Rigorous validation processes, diverse training datasets, and prospective clinical trials.	Continuous improvement of model design and validation methods.
Limited understanding of the causal relationship between retinal biomarkers and neurological diseases in neuro‐ophthalmology.	Interdisciplinary research between ophthalmology, neurology, and other fields.	Enhanced understanding through long‐term studies and multi‐omics approaches.

Abbreviations: AI, artificial intelligence; CONSORT‐AI, consolidated standards of reporting trials‐artificial intelligence; DR, diabetic retinopathy; STARD‐AI, standards for reporting of diagnostic accuracy studies‐artificial intelligence.

## 5 Conclusions

AI has made significant contributions to ophthalmology by addressing critical challenges in disease diagnosis, personalized treatment, and remote care delivery. Despite challenges related to cost, privacy, and interoperability, collaborative efforts among academia, industry, and policymakers are facilitating broader adoption. By harnessing the potential of AI, ophthalmology can achieve a future of equitable, efficient, and personalized patient care.

## Author Contributions

**Chongyang She:** funding acquisition (equal), methodology (equal), writing – original draft (equal). **Yong Tao:** conceptualization (equal), supervision (equal), writing – review and editing (equal).

## Ethics Statement

The authors have nothing to report.

## Consent

The authors have nothing to report.

## Conflicts of Interest

The authors declare no conflicts of interest.

## Data Availability

The data that support the findings of this study are available from the corresponding author upon reasonable request.

## References

[1] L. Dong , W. He , R. Zhang , et al., “Artificial Intelligence for Screening of Multiple Retinal and Optic Nerve Diseases,” JAMA Network Open 5, no. 5 (2022): e229960, 10.1001/jamanetworkopen.2022.9960. 35503220 PMC9066285

[2] T. K. Shah , T. Tariq , R. Phillips , et al., “Health Care for All: Effective, Community Supported, Healthcare With Innovative Use of Telemedicine Technology,” Journal of Pharmaceutical Policy and Practice 11 (2018): 3, 10.1186/s40545-018-0130-5. 29435335 PMC5793345

[3] P. Hamet and J. Tremblay , “Artificial Intelligence in Medicine,” supplement, Metabolism: Clinical and Experimental 69 (2017): S36–S40, 10.1016/j.metabol.2017.01.011. 28126242

[4] U. Sivarajah , M. M. Kamal , Z. Irani , and V. Weerakkody , “Critical Analysis of Big Data Challenges and Analytical Methods,” Journal of Business Research 70 (2017): 263–286, 10.1016/j.jbusres.2016.08.001.

[5] J. M. Nunez do Rio , P. Nderitu , R. Raman , et al., “Using Deep Learning to Detect Diabetic Retinopathy on Handheld Non‐Mydriatic Retinal Images Acquired by Field Workers in Community Settings,” Scientific Reports 13 (2023): 1392, 10.1038/s41598-023-28347-z. 36697482 PMC9876892

[6] V. Gulshan , L. Peng , M. Coram , et al., “Development and Validation of a Deep Learning Algorithm for Detection of Diabetic Retinopathy in Retinal Fundus Photographs,” Journal of the American Medical Association 316, no. 22 (2016): 2402–2410, 10.1001/jama.2016.17216. 27898976

[7] V. Bellemo , Z. W. Lim , G. Lim , et al., “Artificial Intelligence Using Deep Learning to Screen for Referable and Vision‐Threatening Diabetic Retinopathy in Africa: A Clinical Validation Study,” Lancet Digital Health 1, no. 1 (2019): e35–e44, 10.1016/S2589-7500(19)30004-4. 33323239

[8] D. Mahapatra , B. Bozorgtabar , and R. Garnavi , “Image Super‐Resolution Using Progressive Generative Adversarial Networks for Medical Image Analysis,” Computerized Medical Imaging and Graphics 71 (2019): 30–39, 10.1016/j.compmedimag.2018.10.005. 30472408

[9] T. Chen , Y. Bai , H. Mao , et al., “Cross‐Modality Transfer Learning With Knowledge Infusion for Diabetic Retinopathy Grading,” Frontiers in Medicine 11 (2024): 1400137, 10.3389/fmed.2024.1400137. 38808141 PMC11130363

[10] S. M. Waldstein , W.‐D. Vogl , H. Bogunovic , A. Sadeghipour , S. Riedl , and U. Schmidt‐Erfurth , “Characterization of Drusen and Hyperreflective Foci as Biomarkers for Disease Progression in Age‐Related Macular Degeneration Using Artificial Intelligence in Optical Coherence Tomography,” JAMA Ophthalmology 138, no. 7 (2020): 740–747, 10.1001/jamaophthalmol.2020.1376. 32379287 PMC7206537

[11] Q. Yan , D. E. Weeks , H. Xin , et al., “Deep‐Learning‐Based Prediction of Late Age‐Related Macular Degeneration Progression,” Nature Machine Intelligence 2, no. 2 (2020): 141–150, 10.1038/s42256-020-0154-9. PMC7153739 32285025

[12] I. Banerjee , L. de Sisternes , J. A. Hallak , et al., “Prediction of Age‐Related Macular Degeneration Disease Using a Sequential Deep Learning Approach on Longitudinal SD‐OCT Imaging Biomarkers,” Scientific Reports 10 (2020): 15434, 10.1038/s41598-020-72359-y. 32963300 PMC7508843

[13] R. Asaoka , H. Murata , K. Hirasawa , et al., “Using Deep Learning and Transfer Learning to Accurately Diagnose Early‐Onset Glaucoma From Macular Optical Coherence Tomography Images,” American Journal of Ophthalmology 198 (2019): 136–145, 10.1016/j.ajo.2018.10.007. 30316669

[14] F. Li , Y. Su , F. Lin , et al., “A Deep‐Learning System Predicts Glaucoma Incidence and Progression Using Retinal Photographs,” Journal of Clinical Investigation 132, no. 11 (2022): e157968, 10.1172/JCI157968. 35642636 PMC9151694

[15] S. I. Berchuck , S. Mukherjee , and F. A. Medeiros , “Estimating Rates of Progression and Predicting Future Visual Fields in Glaucoma Using a Deep Variational Autoencoder,” Scientific Reports 9 (2019): 18113, 10.1038/s41598-019-54653-6. 31792321 PMC6888896

[16] C. Y. Cheung , H. Li , E. L. Lamoureux , et al., “Validity of a New Computer‐Aided Diagnosis Imaging Program to Quantify Nuclear Cataract From Slit‐Lamp Photographs,” Investigative Ophthalmology & Visual Science 52, no. 3 (2011): 1314–1319, 10.1167/iovs.10-5427. 21051727

[17] X. Wu , Y. Huang , Z. Liu , et al., “Universal Artificial Intelligence Platform for Collaborative Management of Cataracts,” British Journal of Ophthalmology 103, no. 11 (2019): 1553–1560, 10.1136/bjophthalmol-2019-314729. 31481392 PMC6855787

[18] W. Li , Y. Yang , K. Zhang , et al., “Dense Anatomical Annotation of Slit‐Lamp Images Improves the Performance of Deep Learning for the Diagnosis of Ophthalmic Disorders,” Nature Biomedical Engineering 4, no. 8 (2020): 767–777, 10.1038/s41551-020-0577-y. 32572198

[19] Y. Ueno , M. Oda , T. Yamaguchi , et al., “Deep Learning Model for Extensive Smartphone‐Based Diagnosis and Triage of Cataracts and Multiple Corneal Diseases,” British Journal of Ophthalmology 108, no. 10 (2024): e324488, 10.1136/bjo-2023-324488. 38242700 PMC11503034

[20] I. Damian and S. D. Nicoară , “SD‐OCT Biomarkers and the Current Status of Artificial Intelligence in Predicting Progression From Intermediate to Advanced AMD,” Life 12, no. 3 (2022): 454, 10.3390/life12030454. 35330205 PMC8950761

[21] S. Vujosevic , S. J. Aldington , P. Silva , et al., “Screening for Diabetic Retinopathy: New Perspectives and Challenges,” Lancet Diabetes & Endocrinology 8, no. 4 (2020): 337–347, 10.1016/S2213-8587(19)30411-5. 32113513

[22] X. Leng , R. Shi , Z. Xu , et al., “Development and Validation of CNN‐MLP Models for Predicting Anti‐VEGF Therapy Outcomes in Diabetic Macular Edema,” Scientific Reports 14 (2024): 30270, 10.1038/s41598-024-82007-4. 39632987 PMC11618618

[23] A. K. Papazafiropoulou , “Diabetes Management in the Era of Artificial Intelligence,” Archives of Medical Sciences. Atherosclerotic Diseases 9 (2024): 122, 10.5114/amsad/183420. PMC11289240 39086621

[24] P. Romanelli , L. Bianchi , A. Muacevic , and G. Beltramo , “Staged Image Guided Robotic Radiosurgery for Optic Nerve Sheath Meningiomas,” Computer Aided Surgery 16, no. 6 (2011): 257–266, 10.3109/10929088.2011.622615. 21991922

[25] M. Gallardo , M. R. Munk , T. Kurmann , et al., “Machine Learning Can Predict Anti‐VEGF Treatment Demand in a Treat‐and‐Extend Regimen for Patients With Neovascular AMD, DME, and RVO Associated Macular Edema,” Ophthalmology Retina 5, no. 7 (2021): 604–624, 10.1016/j.oret.2021.05.002. 33971352

[26] D. J. Fu , L. Faes , S. K. Wagner , et al., “Predicting Incremental and Future Visual Change in Neovascular Age‐Related Macular Degeneration Using Deep Learning,” Ophthalmology Retina 5, no. 11 (2021): 1074–1084, 10.1016/j.oret.2021.01.009. 33516917

[27] J. M. Han , J. Han , J. Ko , et al., “Anti‐VEGF Treatment Outcome Prediction Based on Optical Coherence Tomography Images in Neovascular Age‐Related Macular Degeneration Using a Deep Neural Network,” Scientific Reports 14 (2024): 28253, 10.1038/s41598-024-79034-6. 39548212 PMC11568167

[28] E. B. Mariottoni , S. Datta , L. S. Shigueoka , et al., “Deep Learning–Assisted Detection of Glaucoma Progression in Spectral‐Domain OCT,” Ophthalmology Glaucoma 6, no. 3 (2023): 228–238, 10.1016/j.ogla.2022.11.004. 36410708 PMC10278200

[29] S. Hussain , J. Chua , D. Wong , et al., “Predicting Glaucoma Progression Using Deep Learning Framework Guided by Generative Algorithm,” Scientific Reports 13 (2023): 19960, 10.1038/s41598-023-46253-2. 37968437 PMC10651936

[30] K. Hou , C. Bradley , P. Herbert , et al., “Predicting Visual Field Worsening With Longitudinal OCT Data Using a Gated Transformer Network,” Ophthalmology 130, no. 8 (2023): 854–862, 10.1016/j.ophtha.2023.03.019. 37003520 PMC10524436

[31] Y. Tian , M. Zang , A. Sharma , S. Z. Gu , A. Leshno , and K. A. Thakoor , “Glaucoma Progression Detection and Humphrey Visual Field Prediction Using Discriminative and Generative Vision Transformers,” in Ophthalmic Medical Image Analysis (Springer Nature Switzerland, 2023), 62–71, 10.1007/978-3-031-44013-7_7.

[32] J. Ladas , D. Ladas , S. R. Lin , U. Devgan , A. A. Siddiqui , and A. S. Jun , “Improvement of Multiple Generations of Intraocular Lens Calculation Formulae With a Novel Approach Using Artificial Intelligence,” Translational Vision Science & Technology 10, no. 3 (2021): 7, 10.1167/tvst.10.3.7. PMC7961112 34003941

[33] O. Zisimopoulos , E. Flouty , I. Luengo , et al., “DeepPhase: Surgical Phase Recognition in CATARACTS Videos,” in Medical Image Computing and Computer Assisted Intervention–MICCAI 2018 (Springer International Publishing, 2018), 265–272, 10.1007/978-3-030-00937-3_31.

[34] N. Cutler , M. Balicki , M. Finkelstein , et al., “Auditory Force Feedback Substitution Improves Surgical Precision During Simulated Ophthalmic Surgery,” Investigative Ophthalmology & Visual Science 54, no. 2 (2013): 1316–1324, 10.1167/iovs.12-11136. 23329663 PMC3597188

[35] J. Jiang , X. Liu , L. Liu , et al., “Predicting the Progression of Ophthalmic Disease Based on Slit‐Lamp Images Using a Deep Temporal Sequence Network,” PLoS One 13, no. 7 (2018): e0201142, 10.1371/journal.pone.0201142. 30063738 PMC6067742

[36] S.‐F. Mohammadi , M. Sabbaghi , H. Z‐Mehrjardi , et al., “Using Artificial Intelligence to Predict the Risk for Posterior Capsule Opacification After Phacoemulsification,” Journal of Cataract and Refractive Surgery 38, no. 3 (2012): 403–408, 10.1016/j.jcrs.2011.09.036. 22244610

[37] N. Ghamsarian , D. Putzgruber‐Adamitsch , S. Sarny , R. Sznitman , K. Schoeffmann , and Y. El‐Shabrawi , “Predicting Postoperative Intraocular Lens Dislocation in Cataract Surgery via Deep Learning,” IEEE Access 12 (2024): 21012–21025, 10.1109/ACCESS.2024.3361042.

[38] E. Long , J. Chen , X. Wu , et al., “Artificial Intelligence Manages Congenital Cataract With Individualized Prediction and Telehealth Computing,” Npj Digital Medicine 3 (2020): 112, 10.1038/s41746-020-00319-x. 32904507 PMC7455726

[39] S. Müller , M. Jain , B. Sachdeva , et al., “Artificial Intelligence in Cataract Surgery: A Systematic Review,” Translational Vision Science & Technology 13, no. 4 (2024): 20, 10.1167/tvst.13.4.20. PMC11033603 38618893

[40] S. L. Mansberger , K. Gleitsmann , S. Gardiner , et al., “Comparing the Effectiveness of Telemedicine and Traditional Surveillance in Providing Diabetic Retinopathy Screening Examinations: A Randomized Controlled Trial,” Telemedicine and e‐Health 19, no. 12 (2013): 942–948, 10.1089/tmj.2012.0313. 24102102 PMC3850428

[41] N. S. Shekhawat , L. M. Niziol , S. S. Sharma , et al., “The Utility of Routine Fundus Photography Screening for Posterior Segment Disease,” Ophthalmology 128, no. 7 (2021): 1060–1069, 10.1016/j.ophtha.2020.11.025. 33253756 PMC8155108

[42] A. Kawaguchi , N. Sharafeldin , A. Sundaram , et al., “Tele‐Ophthalmology for Age‐Related Macular Degeneration and Diabetic Retinopathy Screening: A Systematic Review and Meta‐Analysis,” Telemedicine and e‐Health 24, no. 4 (2018): 301–308, 10.1089/tmj.2017.0100. 28783458 PMC6916253

[43] A. A. Abdulamier , L. M. Shaker , A. A. Al‐Amiery , M. T. Qasim , W. N. R. W. Isahak , and A. A. I. Luthfi , “Advancements and Applications of Smart Contact Lenses: A Comprehensive Review,” Results in Engineering 24 (2024): 103268, 10.1016/j.rineng.2024.103268.

[44] A. Loomba , S. Vempati , N. Davara , et al., “Use of a Tablet Attachment in Teleophthalmology for Real‐Time Video Transmission From Rural Vision Centers in a Three‐Tier Eye Care Network in India: EyeSmart Cyclops,” International Journal of Telemedicine and Applications 2019, no. 1 (2019): 5683085, 10.1155/2019/5683085. 31057606 PMC6463610

[45] TENCON '97 Brisbane – Australia. Proceedings of IEEE TENCON '97. IEEE Region 10 Annual Conference. Speech and Image Technologies for Computing and Telecommunications (Cat. No.97CH36162) (1997), 10.1109/tencon.1997.648201.

[46] R. J. Lyons , S. R. Arepalli , O. Fromal , J. D. Choi , and N. Jain , “Artificial Intelligence Chatbot Performance in Triage of Ophthalmic Conditions,” Canadian Journal of Ophthalmology 59, no. 4 (2024): e301–e308, 10.1016/j.jcjo.2023.07.016. 37572695

[47] M. Savoy , “IDx‐DR for Diabetic Retinopathy Screening,” American Family Physician 101, no. 5 (2020): 307–308. 32109029

[48] A. A. van der Heijden , M. D. Abramoff , F. Verbraak , M. V. van Hecke , A. Liem , and G. Nijpels , “Validation of Automated Screening for Referable Diabetic Retinopathy With the IDx‐DR Device in the Hoorn Diabetes Care System,” Acta Ophthalmologica 96, no. 1 (2018): 63–68, 10.1111/aos.13613. 29178249 PMC5814834

[49] J. Yim , R. Chopra , T. Spitz , et al., “Predicting Conversion to Wet Age‐Related Macular Degeneration Using Deep Learning,” Nature Medicine 26, no. 6 (2020): 892–899, 10.1038/s41591-020-0867-7. 32424211

[50] L. Dai , B. Sheng , T. Chen , et al., “A Deep Learning System for Predicting Time to Progression of Diabetic Retinopathy,” Nature Medicine 30, no. 2 (2024): 584–594, 10.1038/s41591-023-02702-z. PMC10878973 38177850

[51] D. A. Meshram and D. D. Patil , “5G Enabled Tactile Internet for Tele‐Robotic Surgery,” Procedia Computer Science 171 (2020): 2618–2625, 10.1016/j.procs.2020.04.284.

[52] D. Kalogeropoulos , C. Kalogeropoulos , M. Stefaniotou , and M. Neofytou , “The Role of Tele‐Ophthalmology in Diabetic Retinopathy Screening,” Journal of Optometry 13, no. 4 (2020): 262–268, 10.1016/j.optom.2019.12.004. 31948924 PMC7520530

[53] H. Chen , X. Pan , J. Yang , et al., “Application of 5G Technology to Conduct Real‐Time Teleretinal Laser Photocoagulation for the Treatment of Diabetic Retinopathy,” JAMA Ophthalmology 139, no. 9 (2021): 975–982, 10.1001/jamaophthalmol.2021.2312. 34236391 PMC8444028

[54] R. G. Nespolo , D. Yi , E. Cole , D. Wang , A. Warren , and Y. I. Leiderman , “Feature Tracking and Segmentation in Real Time via Deep Learning in Vitreoretinal Surgery,” Ophthalmology Retina 7, no. 3 (2023): 236–242, 10.1016/j.oret.2022.10.002. 36241132

[55] R. G. Nespolo , D. Yi , E. Cole , N. Valikodath , C. Luciano , and Y. I. Leiderman , “Evaluation of Artificial Intelligence‐Based Intraoperative Guidance Tools for Phacoemulsification Cataract Surgery,” JAMA Ophthalmology 140, no. 2 (2022): 170–177, 10.1001/jamaophthalmol.2021.5742. 35024773 PMC8855235

[56] S. Morita , H. Tabuchi , H. Masumoto , T. Yamauchi , and N. Kamiura , “Real‐Time Extraction of Important Surgical Phases in Cataract Surgery Videos,” Scientific Reports 9, no. 1 (2019): 16590, 10.1038/s41598-019-53091-8. 31719589 PMC6851365

[57] T. Wang , J. Xia , R. Li , et al., “Intelligent Cataract Surgery Supervision and Evaluation *via* Deep Learning,” International Journal of Surgery 104 (2022): 106740, 10.1016/j.ijsu.2022.106740. 35760343

[58] E. Rahimy , J. Wilson , T.‐C. Tsao , S. Schwartz , and J.‐P. Hubschman , “Robot‐Assisted Intraocular Surgery: Development of the IRISS and Feasibility Studies in an Animal Model,” Eye 27, no. 8 (2013): 972–978, 10.1038/eye.2013.105. 23722720 PMC3740307

[59] J. T. Wilson , M. J. Gerber , S. W. Prince , et al., “Intraocular Robotic Interventional Surgical System (IRISS): Mechanical Design, Evaluation, and Master‐Slave Manipulation,” International Journal of Medical Robotics and Computer Assisted Surgery 14, no. 1 (2018): e1842, 10.1002/rcs.1842. 28762253

[60] J. W. Kim , C. He , M. Urias , et al., “Autonomously Navigating a Surgical Tool Inside the Eye by Learning From Demonstration,” in 2020 IEEE International Conference on Robotics and Automation (ICRA) (2020), 7351–7357, 10.1109/icra40945.2020.9196537. PMC8493646 34621556

[61] J. W. Kim , P. Zhang , P. Gehlbach , I. Iordachita , and M. Kobilarov , “Towards Autonomous Eye Surgery by Combining Deep Imitation Learning With Optimal Control,” Proceedings of Machine Learning Research 155 (2021): 2347–2358. 34712957 PMC8549631

[62] F. Amore , V. Silvestri , M. Guidobaldi , et al., “Efficacy and Patients' Satisfaction With the ORCAM MyEye Device Among Visually Impaired People: A Multicenter Study,” Journal of Medical Systems 47, no. 1 (2023): 11, 10.1007/s10916-023-01908-5. 36645535

[63] L. C. Olmos de Koo and N. Z. Gregori , “The Argus II Retinal Prosthesis: A Comprehensive Review,” International Ophthalmology Clinics 56, no. 4 (2016): 39–46, 10.1097/IIO.0000000000000144. 27575757

[64] H. Faber , D. Besch , K.‐U. Bartz‐Schmidt , et al., “Restriction of Eye Motility in Patients With RETINA IMPLANT Alpha AMS,” Acta Ophthalmologica 98, no. 8 (2020): e998–e1003, 10.1111/aos.14435. 32304165

[65] Q. Liu , C. Huang , G. Zhan , Y. Guan , and S. Li , “The Need for Expansion of Global Collaborations on AI in Oncology,” Lancet 405, no. 10487 (2025): 1339, 10.1016/S0140-6736(25)00634-8. 40253093

[66] H. Lin , R. Li , Z. Liu , et al., “Diagnostic Efficacy and Therapeutic Decision‐Making Capacity of an Artificial Intelligence Platform for Childhood Cataracts in Eye Clinics: A Multicentre Randomized Controlled Trial,” EClinicalMedicine 9 (2019): 52–59, 10.1016/j.eclinm.2019.03.001. 31143882 PMC6510889

[67] A. Noriega , D. Meizner , D. Camacho , et al., “Screening Diabetic Retinopathy Using an Automated Retinal Image Analysis System in Independent and Assistive Use Cases in Mexico: Randomized Controlled Trial,” JMIR Formative Research 5, no. 8 (2021): e25290, 10.2196/25290. 34435963 PMC8430849

[68] M. D. Abràmoff , P. T. Lavin , M. Birch , N. Shah , and J. C. Folk , “Pivotal Trial of an Autonomous AI‐Based Diagnostic System for Detection of Diabetic Retinopathy in Primary Care Offices,” Npj Digital Medicine 1 (2018): 39, 10.1038/s41746-018-0040-6. 31304320 PMC6550188

[69] T. Pereira , J. Morgado , F. Silva , et al., “Sharing Biomedical Data: Strengthening AI Development in Healthcare,” Healthcare 9, no. 7 (2021): 827, 10.3390/healthcare9070827. 34208830 PMC8303863

[70] J. G. Elmore , C. K. Wells , C. H. Lee , D. H. Howard , and A. R. Feinstein , “Variability in Radiologists' Interpretations of Mammograms,” New England Journal of Medicine 331, no. 22 (1994): 1493–1499, 10.1056/NEJM199412013312206. 7969300

[71] J. G. Elmore , G. M. Longton , P. A. Carney , et al., “Diagnostic Concordance Among Pathologists Interpreting Breast Biopsy Specimens,” Journal of the American Medical Association 313, no. 11 (2015): 1122–1132, 10.1001/jama.2015.1405. 25781441 PMC4516388

[72] M. D. Abràmoff , Y. Lou , A. Erginay , et al., “Improved Automated Detection of Diabetic Retinopathy on a Publicly Available Dataset Through Integration of Deep Learning,” Investigative Ophthalmology & Visual Science 57, no. 13 (2016): 5200–5206, 10.1167/iovs.16-19964. 27701631

[73] R. Gargeya and T. Leng , “Automated Identification of Diabetic Retinopathy Using Deep Learning,” Ophthalmology 124, no. 7 (2017): 962–969, 10.1016/j.ophtha.2017.02.008. 28359545

[74] Z. Li , Y. He , S. Keel , W. Meng , R. T. Chang , and M. He , “Efficacy of a Deep Learning System for Detecting Glaucomatous Optic Neuropathy Based on Color Fundus Photographs,” Ophthalmology 125, no. 8 (2018): 1199–1206, 10.1016/j.ophtha.2018.01.023. 29506863

[75] E. R. Dow , T. D. L. Keenan , E. M. Lad , et al., “From Data to Deployment,” Ophthalmology 129, no. 5 (2022): e43–e59, 10.1016/j.ophtha.2022.01.002. 35016892 PMC9859710

[76] H. Liu , L. Li , I. M. Wormstone , et al., “Development and Validation of a Deep Learning System to Detect Glaucomatous Optic Neuropathy Using Fundus Photographs,” JAMA Ophthalmology 137, no. 12 (2019): 1353–1360, 10.1001/jamaophthalmol.2019.3501. 31513266 PMC6743057

[77] Y. Xu , M. Hu , H. Liu , et al., “A Hierarchical Deep Learning Approach With Transparency and Interpretability Based on Small Samples for Glaucoma Diagnosis,” Npj Digital Medicine 4 (2021): 48, 10.1038/s41746-021-00417-4. 33707616 PMC7952384

[78] X. Liu , S. C. Rivera , L. Faes , et al., “Reporting Guidelines for Clinical Trials Evaluating Artificial Intelligence Interventions Are Needed,” Nature Medicine 25, no. 10 (2019): 1467–1468, 10.1038/s41591-019-0603-3. 31551578

[79] X. Liu , S. Cruz Rivera , D. Moher , et al., “Reporting Guidelines for Clinical Trial Reports for Interventions Involving Artificial Intelligence: The CONSORT‐AI Extension,” Lancet Digital Health 2, no. 10 (2020): e537–e548, 10.1016/S2589-7500(20)30218-1. 33328048 PMC8183333

[80] J. He , S. L. Baxter , J. Xu , J. Xu , X. Zhou , and K. Zhang , “The Practical Implementation of Artificial Intelligence Technologies in Medicine,” Nature Medicine 25, no. 1 (2019): 30–36, 10.1038/s41591-018-0307-0. PMC6995276 30617336

[81] J. P. O. Li , H. Liu , D. S. J. Ting , et al., “Digital Technology, Tele‐Medicine and Artificial Intelligence in Ophthalmology: A Global Perspective,” Progress in Retinal and Eye Research 82 (2021): 100900, 10.1016/j.preteyeres.2020.100900. 32898686 PMC7474840

[82] M. A. Gianfrancesco , S. Tamang , J. Yazdany , and G. Schmajuk , “Potential Biases in Machine Learning Algorithms Using Electronic Health Record Data,” JAMA Internal Medicine 178, no. 11 (2018): 1544–1547, 10.1001/jamainternmed.2018.3763. 30128552 PMC6347576

